# Systematic Study of a Library of PDMAEMA-Based, Superparamagnetic Nano-Stars for the Transfection of CHO-K1 Cells

**DOI:** 10.3390/polym9050156

**Published:** 2017-04-28

**Authors:** Ullrich Stahlschmidt, Valérie Jérôme, Alexander P. Majewski, Axel H.E. Müller, Ruth Freitag

**Affiliations:** 1 Process Biotechnology, University of Bayreuth, Universitätsstrasse 30, 95440 Bayreuth, Germany; bioprozesstechnik@uni-bayreuth.de (U.S.); valerie.jerome@uni-bayreuth.de (V.J.); 2 Evonik Resource Efficiency GmbH, Paul-Baumann-Straße 1, 45772 Marl, Germany; alexander.majewski@evonik.com; 3 Institute of Organic Chemistry, Johannes Gutenberg University Mainz, Duesbergweg 10-14, 55128 Mainz, Germany; axel.mueller@uni-mainz.de

**Keywords:** ATRP, cellular uptake, CHO cells, EGFP, gene delivery, magnetic nanoparticles, PDMAEMA, PDEGMA, polycation, transfection

## Abstract

The introduction of the DNA into mammalian cells remains a challenge in gene delivery, particularly in vivo. Viral vectors are unmatched in their efficiency for gene delivery, but may trigger immune responses and cause severe side-reactions. Non-viral vectors are much less efficient. Recently, our group has suggested that a star-shaped structure improves and even transforms the gene delivery capability of synthetic polycations. In this contribution, this effect was systematically studied using a library of highly homogeneous, paramagnetic nano-star polycations with varied arm lengths and grafting densities. Gene delivery was conducted in CHO-K1 cells, using a plasmid encoding a green fluorescent reporter protein. Transfection efficiencies and cytotoxicities varied systematically with the nano-star architecture. The arm density was particularly important, with values of approximately 0.06 arms/nm^2^ yielding the best results. In addition, a certain fraction of the cells became magnetic during transfection. The gene delivery potential of a nano-star and its ability to render the cells magnetic did not have any correlations. End-capping the polycation arms with di(ethylene glycol) methyl ether methacrylate (PDEGMA) significantly improved serum compatibility under transfection conditions; such nano-stars are potential candidates for future in vivo testing.

## 1. Introduction

The introduction of DNA remains a challenge in genetic modification (‘transfection’) of mammalian cells, in particular for in vivo applications. Much effort has been made over the last decade for the development of effective vectors and molecular gene vehicles [1]. Viral vectors are still unmatched in their efficiency, but trigger immune responses and may show toxic or carcinogenic effects [2]. Non-viral gene vectors, such as synthetic polycations, constitute promising, albeit currently much less efficient, alternatives. In this context, polycations that can be produced with high control over their composition and architecture, are of particular interest for systematic research into underlying structure-function relationships of these molecules [3]. Polycations form stable polyelectrolyte complexes (‘polyplexes’) with polyanionic DNA. Given the right circumstances, mammalian cells take up such polyplexes upon contact with the cellular membrane. The size and charge of the polyplexes significantly influences the uptake mechanism and its efficiency [4,5,6]. A positive net charge will, e.g., facilitate non-specific uptake via endocytosis, a reaction triggered by electrostatic interactions with the negatively charged exterior of the cell [7].

One of the most commonly used polycations for gene delivery is poly(ethyleneimine) (PEI) [8], because of its excellent gene delivery capability and comparatively low production costs. Recently, attention has also been given to poly(dimethylaminoethyl methacrylate) (PDMAEMA) as an alternative, due to its more controllable structure and comparatively low cytotoxicity [9,10,11]. PDMAEMA-based structures have helped to understand fundamental aspects of the structure-function relationship of polycationic gene delivery agents [12]. For instance, increasing molar mass is typically associated with improved gene delivery, but also with increasing toxicity, while for a given molar mass, non-linear polymers such as branched or dendritic polymers show less cytotoxicity with a comparable transfection efficiency [13,14]. In this context, our group has introduced star-shaped, PDMAEMA-based transfection agents (‘nano-stars’) [15], as vectors for gene delivery. The nano-stars combined high transfection efficiencies with low cytotoxicities, while transfecting cells that before had been considered non-susceptible to non-viral gene delivery, including non-dividing, differentiated, or suspension cells [16,17].

In one embodiment, these nano-stars were based on a superparamagnetic maghemite (γ-Fe_2_O_3_) core [18]. Preliminary studies showed that the corresponding polyplexes, but also some of the cells that came into contact with them, became magnetic and were manageable by magnetic fields [18]. The ability to manipulate the polyplexes by magnetic fields opens interesting options, inter alia for medical applications. As shown by others, magnetically guided drug targeting allows non-invasive drug accumulation at specific body locations, with the possibility of administering a more precise dosage of bioactive compounds, while limiting systemic distribution [19]. Since star-shaped polycations have shown the ability to deliver DNA to non-dividing, differentiated, but also suspension cells, they could be considered potential candidates for eventual in vivo applications in the area of DNA medicine.

For this study, a library of highly homogeneous magnetic nano-stars with varied arm length and grafting density was synthesized, including one structure with arms that were end-capped with blocks of poly(diethylene glycol) methyl ether methacrylate) (PDEGMA) for improved blood compatibility. Statistical tools, such as the nonparametric Spearman correlation, were used to correlate physicochemical parameters of both the polycations and the corresponding polyplexes with their eventual ability to deliver plasmid DNA to CHO-K1 cells, and also with their cytotoxicities and magnetic effects.

## 2. Materials and Methods

### 2.1. Materials

Sigma-Aldrich (Taufkirchen, Germany) was used for chemicals, Biochrome AG (Berlin, Germany) and Greiner Bio-One (Frickenhausen, Germany) as suppliers for cell culture materials, media, and solutions, unless otherwise indicated. Culture medium R10 (RPMI-1640 without l-glutamine, from Lonza, Visp, Switzerland) was supplemented with 10% (*v*/*v*) fetal calf serum, 2 mM l-glutamine, and 100 IU/mL penicillin or 100 µg/mL streptomycin prior to use. Culture medium Opti-MEM (reduced serum medium, GlutaMAX™ Supplement, Invitrogen/Life Technology/Thermo Fisher Scientific, Waltham, MA, USA) was used as received from the supplier (Invitrogen/Life Technology/Thermo Fisher Scientific, Waltham, MA, USA). Dulbecco’s Phosphate-Buffered Saline (DPBS) without Ca^2+^ and Mg^2+^ (Lonza, Visp, Switzerland) was used. NaCl (150 mM in Milli-Q water) and HEPES buffered glucose (HBG, 20 mM HEPES, 5 wt % glucose, pH 6.0) solutions were prepared in house and sterilized by filtration (0.2 µm, cellulose acetate filters).

### 2.2. Cells

Chinese Hamster Ovary-K1 (CHO-K1) cells (CCL-61, ATCC) were maintained as recommended by the supplier in R10 culture medium.

### 2.3. Plasmid

pEGFP-N1 (4.7 kb, Clontech Laboratories, Inc., Mountain View, CA, USA) encoding for the enhanced green fluorescent protein (EGFP) driven by the cytomegalovirus (CMV) immediate early promoter was used as a reporter. pEGFP-N1 was amplified in *E. coli* DH5α using standard laboratory techniques and purified by the EndoFree Plasmid Kit (Giga Prep) from QIAGEN (Hilden, Germany). Quality control: >80% supercoiled topology (agarose gel) and A_260_/A_280_ ≥ 1.8. Purified plasmids were solubilized in PCR-grade water and stored at −20 °C.

### 2.4. Transfection Agents

The nano-stars are listed in Table 1. Synthesis of the oleic acid maghemite (γ-Fe_2_O_3_) core was as previously described by Majewski et al. [15]. Briefly, a 500 mL two-necked round-bottom flask, connected to a reflux condenser, was charged with 250 mL dioctyl ether and 58 mL oleic acid (51.52 g, 182.4 mmol), and degassed with nitrogen for 15 min. The reaction mixture was heated to 100 °C under nitrogen atmosphere before adding Fe(CO)_5_ (8 mL, 60.8 mmol). Subsequently, the mixture was heated to reflux for 1.5 h until the color of the solution turned black. After cooling to room temperature, the reaction mixture was stirred under air to initiate the oxidation process to γ-Fe_2_O_3_. The nanoparticles were precipitated with ethanol and collected by a NdFeB magnet. After decantation of the supernatant, the nanoparticles were redispersed in toluene, followed by further purification via precipitating with ethanol, magnetic collection, and redispersion in cyclohexane (repeated three times) yielding the final stock solution (8 g/L γ-Fe_2_O_3_@oleic acid in cyclohexane).

Subsequently, the particles were coated in a single step by a thin silica shell functionalized with an atomic transfer radical polymerization (ATRP) initiator, as also described previously [20]. For this, a 500 mL round-bottom flask was charged with 8.2 g Igepal^®^ (Sigma-Aldrich, Taufkirchen, Germany) CO-520 (polyoxyethylene(5)nonylphenyl ether, 18.6 mmol) and 250 mL cyclohexane. The reaction mixture was incubated in an ultrasonic bath for 10 min to dissolve the Igepal^®^ CO-520. Subsequently, 6.25 mL of the γ-Fe_2_O_3_@oleic acid stock-solution were dispersed, before adding 1.43 mL of a 25% aqueous ammonia solution (formation of a brownish reverse microemulsion). The reaction was commenced by adding 0.36 mL TEOS (1.6 mmol) and placing the reaction mixture in an incubation shaker at 100 rpm for 48 h at ambient temperature. Between 30 µL and 150 µL of 2-bromoisobutyryl-6-(trichlorosilyl)hexanoate (BIBSI) were added for functionalization, and the reaction mixture was placed back in the shaker for 24 h. The cyclohexane was removed by a rotary evaporator, and the mixture was dialysed against acetone to remove water and non-bound initiator.

PDMAEMA arms were grown from the core particles via ATRP [21]. Here the protocol for P1 is given (75 µL BIBSI for functionalization). For the other nano-stars this general protocol was modified as indicated in Table 2. A 50 mL screw cap glass equipped with a septum was charged with 40 mg silica-shell coated nanoparticles dispersed in 20 mL acetone, 20 mL DMAEMA (18.7 g, 118.7 mmol), and 3 mg CuCl (0.03 mmol). The mixture was purged with nitrogen for 30 min before adding 8 µL degassed 1,1,4,7,10,10-hexamethyl triethylenetetramine (HMTETA, 7 mg, 0.03 mmol) dissolved in 2 mL acetone. The reaction mixture was heated to 60 °C for 4 h. After cooling down to room temperature, the reaction was terminated by exposing the agitated mixture to air for 10 min. The crude product was purified by several cycles of centrifugation/redispersion in methanol to remove the copper catalyst and remaining monomers. The grafted hybrid particles were finally redispersed in acetonitrile.

In addition, one version of nano-stars were synthesized with PDMAEMA arms end-capped with PDEGMA blocks, using a second ATRP step. Briefly, a 250 mL screw cap glass with septum was charged with 280 mg PDMAEMA-grafted nanoparticles dispersed in 100 mL acetonitrile, 115 mL DEGMA (117.3 g, 623.2 mmol), and 10 mg CuCl (0.1 mmol). The mixture was purged with nitrogen for 30 min before adding 27 µL degassed HMTETA (23 mg, 0.1 mmol) dissolved in 2 mL acetonitrile. The reaction mixture was heated to 60 °C for 48 h. Termination of the reaction and subsequent work-up was performed as described above for the PDMAEMA nano-stars. The grafted hybrid particles were finally redispersed in deionized water.

All nano-stars were characterized after synthesis by thermogravimetric analysis (TGA) to calculate the number and length of the PDMAEMA arms as previously described [18]. The core diameter was determined by transmission electron microscopy and size exclusion chromatography. Magnetic properties including magnetization curves were as published previously [18], and typical of superparamagnetic particles. Values were similar in all cases, since silica encapsulation and subsequent polymerization have no significant influence on superparamagnetic behavior. 

### 2.5. Transfection Protocol

Adherent CHO cells were harvested by trypsinization (standard laboratory protocol including trypsin inactivation by R10), and seeded 24 h before transfection at a density of 4 × 10^5^ cells in 2 mL R10 in 6-well plates. One hour before transfection, cells were rinsed with DPBS and covered with 1 mL Opti-MEM. Unless indicated otherwise, polyplexes were prepared by mixing 1 µg DNA into 50 µL of aqueous 150 mM NaCl solution, then adding 1 mL Opti-MEM, followed by the necessary amount of polycation stock solution (single drop) to reach the desired N/P-ratio (N nano-star/P DNA, calculation, see below). Mixtures were vortexed for 10 s and incubated for 30 min at room temperature, then added to the cells. The plates were then placed for three to four hours in a cell culture incubator, and the cells were subsequently transferred to fresh R10 medium for further cultivation/analysis. All transfection experiments were performed in duplicate.

### 2.6. N/P Ratio Calculation

N/P-ratios were calculated according to:

(1) $$\text{Number of equivalent}=\frac{µL\;\text{polycation stock solution}*\left[N\right]}{µg\;\mathrm{pDNA}*3}$$

with [*N*] = concentration of nitrogen residues in mM.

### 2.7. Analysis of the Transfection Efficiency by Flow Cytometry

Cells were harvested by trypsinization and pooled with the original culture supernatant to include detached (mitotic, dead) cells from the culture. After a centrifugation step (200 *g*, 5 min), cells were resuspended in 1 mL DPBS containing 1 μg/mL propidium iodide (PI) to counterstain the dead cells, and analyzed using a Cytomics FC500 flow cytometer (Beckman Coulter, Krefeld, Germany) equipped with a 488 nm argon-ion laser. Data were collected from at least 100,000 events. The total cell population was analyzed for red fluorescence (620 nm, PI) to determine the overall viability. Cells were then evaluated by scatter properties (FSC/SSC) to select a region representing single, non-apoptotic cells (elimination of dead cells, debris and cellular aggregates). The relative EGFP fluorescence (525 nm) of the gated cells was measured, allowing a statistical quantification of the percentage of EGFP expressing cells (‘transfection efficiency’) in the non-apoptotic cell population. Transfected cells were further subdivided into high (>100 a.u.), middle (10–100 a.u.), and low (1–10 a.u.) producers.

### 2.8. Determination of the Magnetic Cell Fraction

Cells were harvested by trypsinization and recovered by centrifugation (200 *g*, 5 min). The cell pellet was resuspended in 1.4 mL RPMI 1640, transferred into a 1.5 mL Eppendorf tube, and put into a 16-Tube SureBeads™ Magnetic Rack (Bio-Rad, Munich, Germany), which was refrigerated overnight. The non-magnetic cell fraction (supernatant) was then collected and transferred into a fresh tube. Loosely attached or sedimented cells were recovered by washing the bottom of the tube in the magnetic rack twice with 15 µL DPBS and adding this to the original supernatant. Magnetic cells were recovered after removal of the tube from the magnetic rack by resuspension in 1.4 mL DPBS. Cell densities in both suspensions were determined using a Vi-Cell cell counter (Beckman Coulter, Krefeld, Germany).

### 2.9. Magnetically Assisted Cell Sorting

The Magnetically-assisted-cell sorting (MACS) system from Miltenyi Biotec (Bergisch Gladbach, Germany) was used according to the manufacturer’s instructions. Cells were harvested by trypsinization and recovered by centrifugation (200 *g*, 5 min). The cell pellet was resuspended in 500 µL of ‘column buffer’ and applied to the column. Afterwards, the column was washed three times with 3 mL of column buffer and the effluent was collected (‘non-magnetic cell fraction’). Then the column was removed from the separator and the magnetic cell fraction recovered by flushing the column with 5 mL of column buffer. Cells from both fractions were recovered by centrifugation (200 *g*, 5 min) and resuspended in 5 mL DPBS each. Cell densities in both fractions were determined using a Vi-Cell cell counter.

### 2.10. Zeta Potential and Hydrodynamic Radius

Zeta potentials and hydrodynamic radii of the polyplexes were determined using a Zetasizer Nano ZS (Malvern, Herrenberg, Germany). Polyplexes were prepared as for transfection, and transferred into folded capillary cells (Malvern, Herrenberg, Germany). After 30 min of incubation at room temperature, the zeta potentials were determined by Laser Doppler Micro-Electrophoresis applying the M3-PALS (Phase Analysis Light Scattering) technique. The size of the formed polyplexes was determined by Non-Invasive Back Scattering (NIBS) utilizing a He-Ne laser (*λ* = 633 nm, max = 5 mW).

### 2.11. DNA Melting Temperature Assay (ΔT-Assay)

One microgram of DNA was mixed with SYBR green I (final concentration: 2.5 × 10^–5^ of stock solution) and incubated in a final volume of 50 µL of either 150 mM NaCl or HBG solution for 15 min on ice. Enough polycation stock solution was added to the reaction mixture to reach the desired N/P-ratio. The mixture was vortexed for 10 s and incubated for 30 min at room temperature in the dark. The sample was then placed in Real Time tubes in a Real Time PCR machine (MX3005P, all Agilent Technologies, Waldbronn, Germany) equipped with an SYBR green filter (BP 504 ± 12 nm). The temperature was increased from 25 to 95 °C at a rate of 1 °C/min. The melting temperature of the DNA was defined as the inflection point of the sigmoidal curve of the relative SYBR green fluorescence (fluorescence at a given temperature relative to the fluorescence at the starting temperature *T* = 25 °C) as a function of the temperature.

### 2.12. Statistical Analyses

Group data are reported as mean ± S.E.M. One-way ANOVA (Bonferroni *t*-test for pairwise comparison) was used to determine whether data groups differed significantly from each other. Unless indicated, statistical significance was defined as *p* < 0.05. A number of flow cytometry experiments were reported where large numbers of cells were evaluated to yield what amounts to a single data point. The experiments were carried out at least in duplicate and in such cases, the indicated deviation represented the average value of the duplicates, together with the random experimental error of the measurement itself. For statistical analysis, R software version 3.3.1 (R Foundation for Statistical Computing, Vienna, Austria) with packages Hmisc version 3.17-4, Corrplot version 0.77, and RColorBrewer version 1.1.2, was used [22]. The correlation coefficient, rho, was calculated according to the nonparametric Spearman correlation statistic test and used to estimate correlations between data sets. Rho values between 0 and 0.25 were defined as no correlation, 0.25 to 0.5 as weak, 0.5 to 0.75 as medium and 0.75 to 1 as a strong correlation. The rho values were plotted in a correlogram. Data points where *p* > 0.01 were considered to show no differences of statistical significance, and were therefore excluded from the correlations.

## 3. Results

### 3.1. Size and Surface Charge of the Polyplexes as a Function of the N/P-Ratio

**Figure 1:**
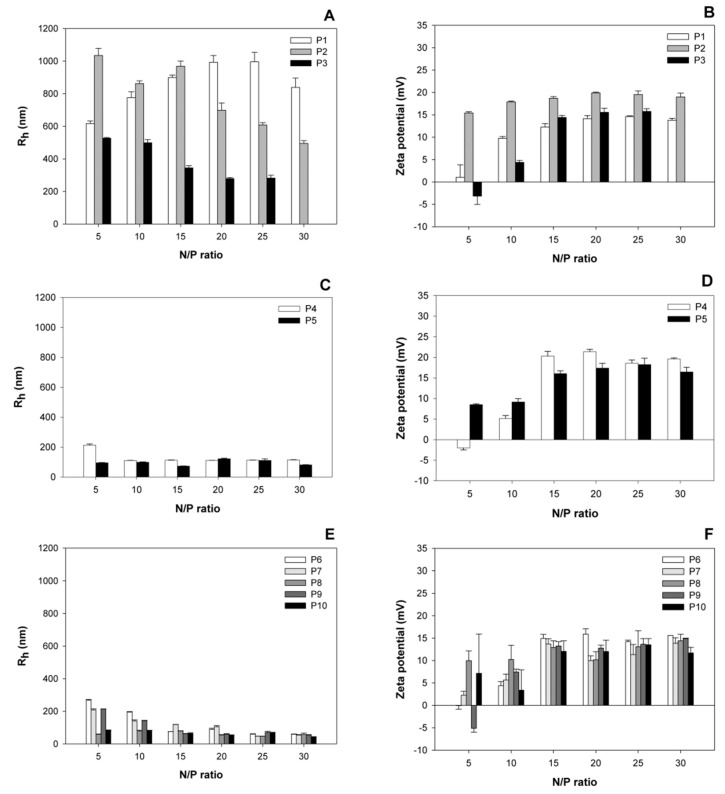
Average hydrodynamic radii (*R*_h_) and zeta potentials of polyplexes prepared at the indicated N/P ratio. Data represent mean ± S.E.M., *n* ≥ 3.

Polyplexes covering a wide range of N/P-ratios were prepared and analyzed for size and surface charge (zeta potential) after a 30 min incubation (Figure 1). Based on the hydrodynamic radii of the corresponding polyplexes, the investigated nano-stars could be divided into three subgroups. Nano-stars with a low arm density, namely P1–P3, formed large polyplexes with sizes around 1000 nm. The effect was most pronounced for P1 (0.006 arm per nm^2^) and P2 (0.011 arm per nm^2^), while the behavior of the polyplexes formed with P3 (0.024 arm per nm^2^) already approached that of the polyplexes formed with the middle arm density nano-stars P4 (0.054 arm per nm^2^) and P5 (0.064 arm per nm^2^). Middle, but also high arm density nano-stars (P6–P10) formed smaller polyplexes with hydrodynamic radii ≤ 300 nm, a size that is considered suitable for endocytosis and transfection [23]. The molar mass of the nano-star, on the other hand, had little influence on the hydrodynamic radius of the ensuing polyplex, for example, polyplexes formed with P10 (*M*_n_ 172,466 kg/mol) were smaller than those formed with P5 (*M*_n_ 12,470 kg/mol).

In terms of the development of size and zeta potential as a function of the N/P-ratio, the zeta potential of all polyplexes increased with the N/P-ratio, and stabilized for N/P-ratios ≥ 15 at values between 15 and 22 mV. No statistically relevant differences were observed between the plateau values of the different nano-stars. Similar values, i.e., maximum zeta potentials around 20 mV, have previously been reported for other polycationic transfection agents [23]. Most polyplexes were already positively charged at the lowest investigated N/P-ratio, i.e., N/P 5. Only P3, P4, and P9 formed exceptions. In their case, the surface charge of the corresponding polyplexes became positive for N/P ≥ 10. 

The development of the hydrodynamic radii as a function of the N/P-ratio was again different for the three nano-star subgroups. Polyplexes that formed with the high arm density nano-stars (P6 to P10) became smaller with increasing N/P-ratio. The main reduction in polyplex size was seen in the N/P-range, where the surface charge (zeta potential) of the polyplexes was still increasing. Polyplexes formed with the middle arm density nano-stars P4 and P5, showed little dependency on the N/P-ratio, while the size of the large polyplexes formed with nano-stars P1 and to some extent also P2 passed through a maximum as a function of the N/P-ratio. 

The formation of large polyplexes as seen here in particular for P1 and P2, has been observed before, and is typically caused by aggregation during complex maturation [24,25,26]. At present, it is not clear why the nano-stars with lowest arm densities yielded the largest polyplexes, i.e., showed the highest aggregation tendency. It is possible that due to the lower arm (and hence charge) density the repulsive forces between individual polyplexes are smaller, which would facilitate aggregation. The reduction in polyplex size with the N/P ratio, as observed in particular for nano-stars P6 to P10, but also P3 as well as P1/P2 beyond the size maximum, may then be due to the fact that the surface net charge increases with increasing N/P-ratios, which would increase the electrostatic repulsion between the individual polyplexes and make aggregation less likely. Moreover, another possible cause for aggregation is the bridging of several polyplexes by individual DNA molecules [18]. The occurrence of unsaturated individual DNA molecules, and in consequence bridging, also becomes less likely at increasing N/P-ratio. 

### 3.2. Melting Temperatures of Polyplexed DNA as Indication of Polyplex Stability

Binding of the polycation to the negatively charged DNA-backbone weakens the DNA double helix, which results in a measurable decrease in the DNA melting temperature (*T*_M_). Lower *T*_M_ values thus indicate stronger polyelectrolyte interaction, and consequently a higher polyplex stability. The DNA melting temperature assay (∆*T*-assay) based on this phenomenon has previously been proposed by us as indirect measure for polyplex stability [27].

**Figure 2:**
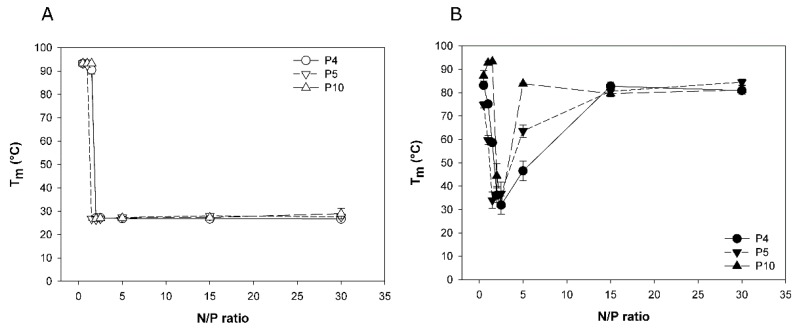
DNA melting temperature (*T*_M_) as function of the N/P-ratio (**A**) polyplexes prepared in HBG, (**B**) polyplexes prepared in aqueous 150 mM NaCl solution. Lines serve as guides. Data represent mean ± S.E.M., *n* ≥ 2. Standard deviations are given for all data points but may be too small for viewing.

Three nano-stars were tested in the *∆T*-assay, two with a middle arm grafting density (P4: 0.054 arms per nm², P5: 0.064 arms per nm²) and one with a high arm density (P10; 0.300 arms per nm²). Nano-stars with low arm grafting density were not tested, since the size of the corresponding polyplexes was considered too big for efficient transfection. *T*_M_ was measured for two standard polyplexation matrices, namely HBG and 150 mM NaCl, and results are compiled in Figure 2.

When formed in HBG, the *T*_M_-data of all three investigated nano-stars were similar and indicative of strong binding. *T*_M_ dropped rapidly from around 90 °C (free DNA) to approximately 27 °C for any N/P-ratio ≥ 2. In case of an aqueous NaCl solution as matrix, a similar decrease in *T*_M_ was seen for an N/P-ratio of 2. However, with further increase of the N/P-ratio, the *T*_M_ increased again, approaching the *T*_M_ of free DNA. An increasing arm density seemed to promote this behavior. We propose a pH-effect to explain the observation. The PDMAEMA forming the arms of the nano-stars is a weak polymeric base. Its degree of protonation and hence its charge density would depend strongly on the matrix pH.

**Figure 3:**
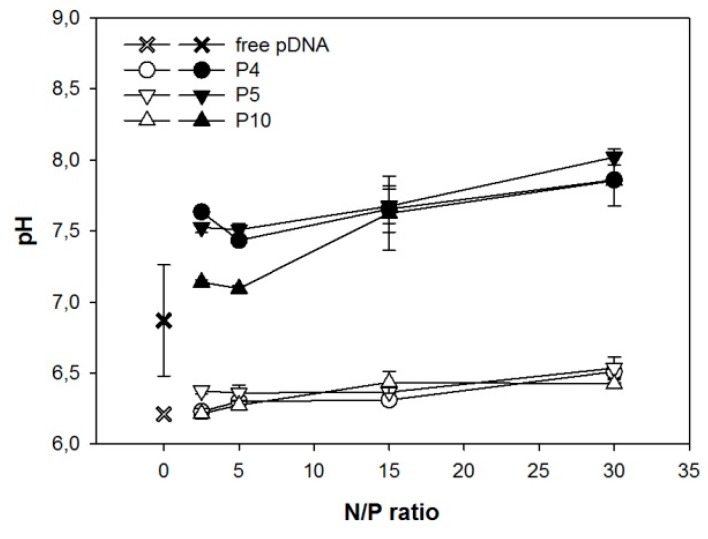
pH of the polyplex solution as a function of the N/P-ratio. Polyplexes were prepared either in HBG (empty symbols) or aqueous 150 mM NaCl solution (filled symbols). Lines serve as a guide. Data represent mean ± S.E.M., *n* ≥ 2.

The development of the pH in the investigated matrices is shown in Figure 3. In the buffered HBG-matrix, the pH showed little change with the N/P-ratio. Consequently, there was no change in the degree of protonation, and hence the charge density of the PDMAEMA. The corresponding polyplexes remained stable. In case of the unbuffered NaCl-matrix, the pH changed from 6.8 in case of the free DNA, to approximately 8 at N/P 30. At pH 8, the degree of protonation of the PDMAEMA would be less than 50% [28], leading to a corresponding reduction in polyplex stability. The degree of protonation of a polycation is in addition influenced by the local charge density. An increasing arm density should therefore result in a lower degree of protonation, and consequently less stable polyplexes under otherwise similar conditions. This would explain the observed effect of the arm density on the stability of complexes formed in an aqueous NaCl solution as matrix.

HBG has been suggested by van Gaal et al. as a matrix for polyplex formation, and has been reported to result in more efficient transfections than with NaCl [4,16]. This has been ascribed to the fact that complexes formed in HBG are smaller and show a smaller tendency for aggregation during incubation. However, as shown in Figure 1, for the N/P-ratios typical for transfection, polyplex aggregation was not a problem, even when using NaCl solution as a matrix. We therefore propose that it is the degree of protonation and in consequence the polyplex’s stability rather than its size that is affected when the standard NaCl matrix is replaced by a buffered medium such as HBG, and that this plays a decisive role in determining transfection efficiency and also cytotoxicity.

### 3.3. Uptake of the Polyplexes by CHO-K1 Cells

**Figure 4:**
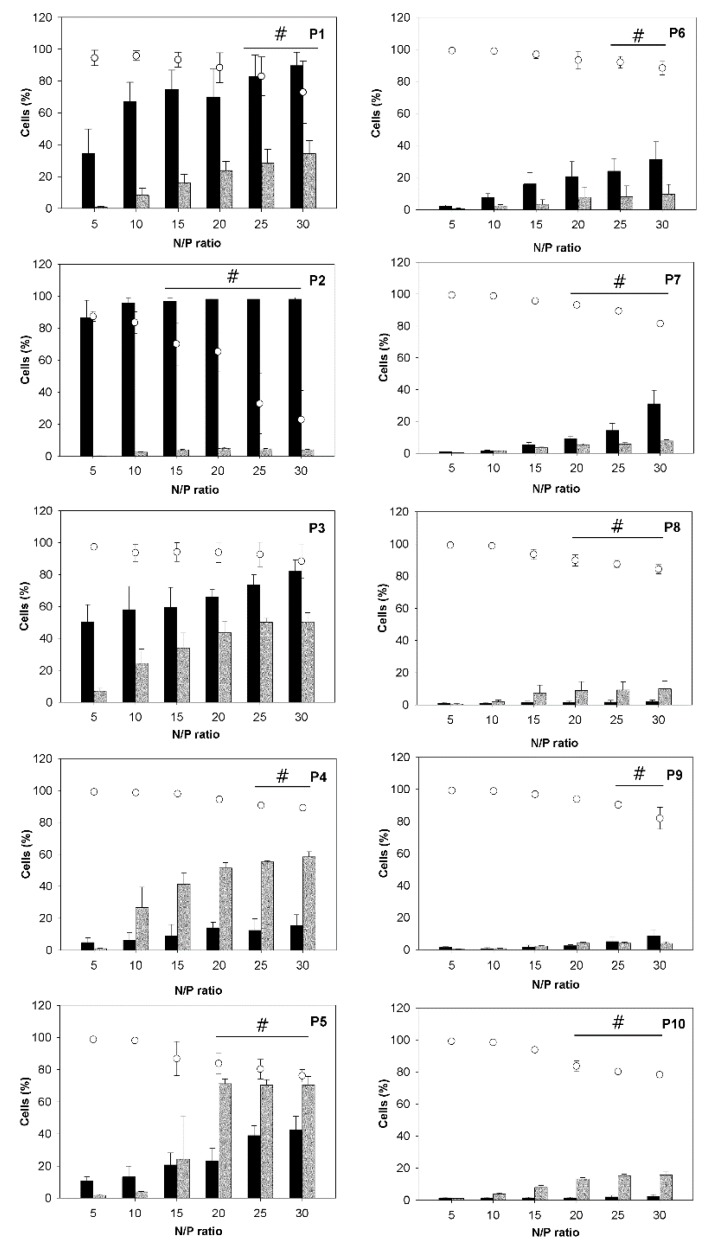
Transfection efficiencies (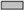), magnetic cell fractions (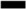), and viabilities (○) determined 26 h post transfection. Data represent mean ± S.E.M., *n* ≥ 3. In the case of the viabilities, statistically significant differences (*p* < 0.05) compared to mock transfected cells (97.6% ± 2.5%, *n* ≥ 30) are denoted by #.

The polyplexes were subsequently used to transfect CHO-K1 cells. Figure 4 summarizes transfection efficiencies (percentage of cells expressing the green fluorescent protein), viabilities, and the fraction of magnetic cells, determined 26 h after exposure of the cells to the polyplexes. Transfection efficiencies and magnetic cell fractions increased with the N/P-ratio, while viabilities decreased. While the trend of decreasing viability was observable for all investigated nano-stars, in most cases, the decrease was small and of no statistical significance in some cases. This general development mirrored the behavior previously documented for other polycationic transfection agents, including PEI, and was directly linked to the increasing amount of surplus polycation present in the transfection mix. Free polymer chains promote internalization and thereby aid transfection, but they also destabilize the membrane and therefore are toxic [29].

Trends observed as a function of the polymer architecture, in particular the arm density, were less simple. The clearest trend was seen for the fraction of the magnetic cells, which tended to decrease with increasing arm density. However, it was not clear whether the cells needed to actually ingest the nano-stars/polyplexes to become magnetic. It was possible that the polyplexes were attaching to the outside of the cells without triggering endocytosis/cellular uptake. Simple attachment should be more likely for larger polyplexes, and in this context it is interesting to note that those nano-stars in particular that formed polyplexes with large hydrodynamic radii (P1, P2) resulted in a disproportionally high percentage of magnetic cells, while their transfection efficiency was rather low.

The optimum transfection efficiency was generally a function of the arm density. Nano-stars with grafting densities between 0.024 chains/nm² (P3) and 0.064 chains/nm² (P5) gave the best transfection results. The highest individual value was found for P5 (71% ± 3% EGFP-positive cells) at N/P-ratios ≥ 20. The comparative inability of the nano-stars with lower arm density (P1, P2) to transfect the cells can be explained by the large size of the corresponding polyplexes, which inhibited cellular uptake through endocytosis. This was not the cause of the low transfection efficiencies observed for the high arm density nano-stars (P6–P10). An increase in cytotoxicity was also unlikely, since only a weak, though statistically significant decrease in viabilities was observed for these nano-stars. At present, we hypothesize that the lower transfection ability of the high arm density nano-stars is related to polyplex stability, which became reduced at higher grafting density, as discussed in Subsection 3.2.

### 3.4. Correlation between Magnetism and Transfection Efficiency

**Figure 5:**
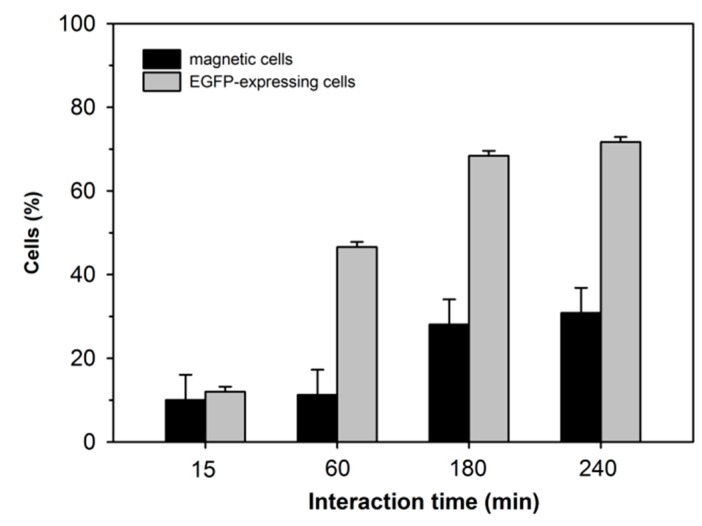
Influence of contact time on transgene-expressing and magnetic cell fractions. Cells were transfected with P4 at N/P 15. Data represent one experiment carried out in duplicate, with random experimental error shown.

As shown, the gene delivery potential of a given nano-star and its ability to render the cells magnetic did not necessarily correlate. As shown in Figure 5 for cells transfected with P4, the respective developments of the percentage of transfected vs. magnetic cells differed also in their dependency on the contact time with the polyplexes (note: 240 min corresponds to the standard incubation period prescribed in the transfection protocol).

**Figure 6:**
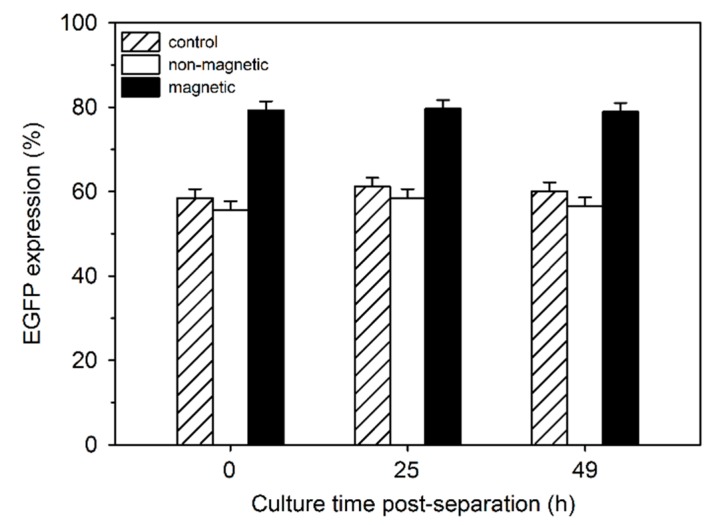
Enhanced green fluorescent protein (EGFP) expression in magnetic and non-magnetic cell fractions compared to the controls (no separation). Cells were transfected with P4 (N/P 20), separated 24 h post transfection (*t* = 0) by magnetically-assisted cell sorting and placed into separated cultures. Data represent one experiment carried out in duplicate, with random experimental error shown.

For a more detailed investigation into the relationship between EGFP expression and magnetism, cells were transfected using P4 (N/P 20) and separated by magnetically-assisted cell sorting after 24 h. Both fractions were separately put back into culture. A non-separated aliquot from the same culture served as a control. EGFP expression by the cells from the two fractions was compared to that of the controls directly after separation as well as 25 and 49 h later (Figure 6). During cell separation, about 20% of the cells were lost, i.e., presumably they never eluted from the column. This was supported by the fact that the viability of the magnetic cell fraction was below 70% directly after separation. After 24 h in culture, the viability again reached >90%.

Compared to the controls, EGFP expressing cells were consistently more frequent in the magnetic cell fraction, while the non-magnetic fraction was slightly depleted. Moreover, when the EGFP-expressing cells were further divided into low, middle and high producers, a slight but statistically significant shift to the high producers was observed in the magnetic cell fraction (Table 3).

**Figure 7:**
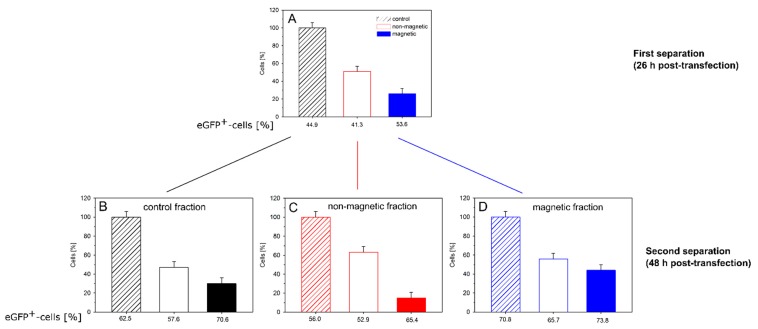
EGFP expression in the respective magnetic, non-magnetic and control cell cultures. Initial separation by magnetic cell sorting took place 26 h after transfection (P4, N/P = 20). The individual cultures were again sorted 22 h later. Data represent one experiment carried out in duplicate with the random experimental error.

Finally, we examined the persistence of magnetism (and transgene expression) within the different cell fractions. For this purpose, CHO-K1 cells were transfected (P4, N/P = 20) and cultured/separated as before. The magnetic and non-magnetic cell fractions, as well as a non-separated aliquot from the same culture, were placed into culture for another 22 h. Afterwards, all three cultures were again subjected to magnetic cell sorting (Figure 7).

The first separation (*t* = 0, 26 h after transfection) gave a similar result as before (Figure 7A). About half the cells were non-magnetic; approximately 30% magnetic and approximately 20% of the cells were lost during sorting. We presume that mainly magnetic cells were lost, i.e., they did not elute from the column, which would make up the balance. The magnetic cell fraction again showed a slightly higher fraction of EGFP-expressing cells than the non-magnetic fraction. However, while statistically relevant, the difference was not very pronounced. When cells from the non-separated control culture were sorted for the second time 22 h later (i.e., 48 h post transfection), the relative distribution over magnetic and non-magnetic cells was similar, while the absolute transfection efficiencies had increased by approximately 10% (Figure 7B). Such an increase in the fraction of transfected cells can be explained by the time required to build up the expression of the transgene.

When the magnetically separated cultures were reexamined 48 h post transfection, the “non-magnetic” fraction from the initial separation (now “control” in Figure 7C) showed a transfection efficiency that was almost identical to that of the non-magnetic fraction obtained from the control culture during the second cell sorting. When the “non-magnetic” cells were subjected to magnetic cell sorting for a second time, a small fraction (15%) of magnetic cells was collected, whose transfection efficiency was significantly higher than that of the bulk, while the fraction of cells (63%) collected once more as “non-magnetic” during the second sorting showed a somewhat lower transfection efficiency than the bulk (Figure 7C). It is not clear how the magnetism was caused in a significant fraction of the cells that had not adhered to the magnetic column during the first sorting. However, the correlation between magnetism and transgene expression was maintained.

In case of the cells from the magnetic fraction produced during the first sorting (Figure 7D), two things were observed. First, there was little additional loss of cells during sorting; close to 100% of the cells were recovered. A total of 56% of the cells had lost their magnetic properties, these cells also contained a lower fraction of transgene expressers, while 44% of the cells were still magnetic and expressed EGFP in large numbers.

Between the moment of transfection and the second cell sorting 48 h later, the cells had, on average, passed through two cycles of cell division. Approximately 50% of magnetic cells in the first cell sorting after 26 h, could thus be explained by a dilution during cell division. However, the fraction of magnetic cells obtained in the second sorting after 48 h should then have been much lower. Instead, we propose that the polymers were distributed to both daughter cells during cell division. In this context, it should be noted that even after release of the delivered DNA from the polyplexes, sufficient amounts of polyanionic molecules (RNA, proteins) would be found in the cytosol to form polyelectrolyte complexes with the polycations, which would then assist in the maintenance of the cells’ magnetic character. Incidentally, this could also be considered circumstantial evidence against an active expulsion of the nano-stars from the cells.

### 3.5. Statistical Analysis of the Correlation

**Figure 8:**
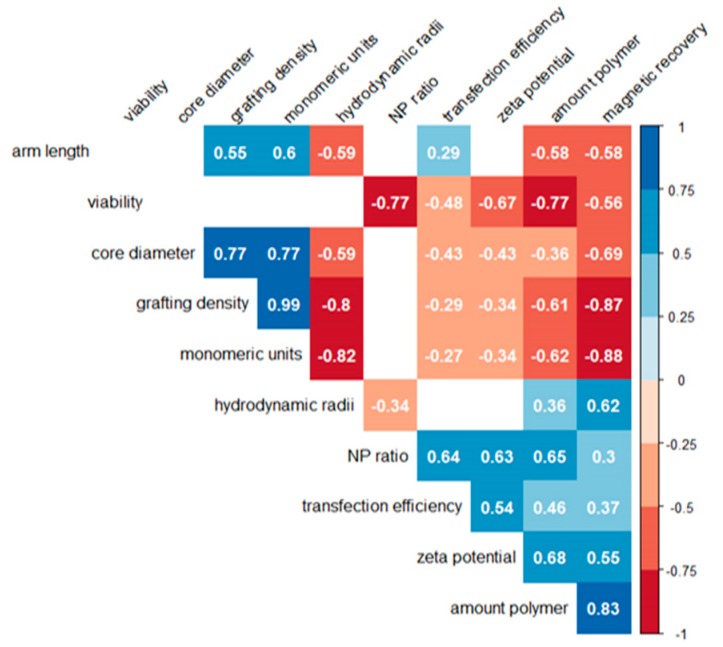
Correlogram between nano-star/polyplex characteristics and the cellular responses (viability, transfection efficiency, magnetism). Positive correlations are indicated in blue, negative ones in red. No correlation coefficients were calculated for parameters, which showed no statistically relevant differences (*p* > 0.01).

A nonparametric Spearman correlation analysis was used to statistically evaluate possible links between the molecular characteristics of the nano-stars (core diameter, arm density, arm length, number of monomeric units per nano-star), the physicochemical properties of the corresponding polyplexes (hydrodynamic radius, zeta potential), the transfection conditions (N/P ratio, amount of polymer), and the cellular reactions (transfection efficiency, magnetism, viability). The resulting correlogram is shown in Figure 8.

Amongst the cellular parameters, a positive correlation was found between transfection and magnetism, which both correlated negatively with viability. Neither of these correlations was very strong. In particular, we looked for (positive) correlations between nano-star and/or polyplex characteristics and the transfection efficiency, since this would be valuable for future optimization attempts. However, except for a medium-strength positive correlation to the N/P-ratio and the zeta potential, both of which were already known, there was only a weak negative correlation to the core diameter of the nano-star. All other parameters, such as arm length, arm density, molar mass, or hydrodynamic radius showed no statistically relevant links to the transfection efficiency. Adding more polymer to the well for a given amount of DNA also aided transfection, however this correlation was weak (0.48) and has been described before, if only qualitatively [16].

Viability, on the other hand, showed a strong negative correlation (−0.77) with the N/P-ratio and the amount of added polymer (–0.77). This enforces a conclusion drawn in a previous paper, that N/P-ratios in transfection should not be adjusted via adding more polymer, but rather by keeping the polymer concentration at a biocompatible level and adjusting (reducing) the amount of added DNA [16]. According to the values presented in Figure 8, transfection efficiencies would not be affected greatly, while viabilities should benefit from this approach. Very strong positive correlations were also found between cellular magnetism and the amount of added polymer (0.83), while the correlation with the arm density (−0.87) and the nano-star mass (−0.88) was negative. The arm length was less relevant, even though this correlation was also negative (−0.58).

### 3.6. Performance of the PDEGMA-Capped Nano-Stars

Magnetic PDMAEMA-based nano-stars may become the starting point for the development of non-viral, synthetic transfection agents with potential for medical applications. However, the blood compatibility of the simple polycations would be too low. In an attempt to improve the blood compatibility of the structures, a nano-star (DB) was produced, with PDMAEMA-arms end-capped with PDEGMA blocks (Table 1). Others have shown that this can lower cytotoxicity and lead to higher transfection efficiencies in the presence of serum, compared to structurally similar, but unmodified polymers [30,31].

**Figure 9:**
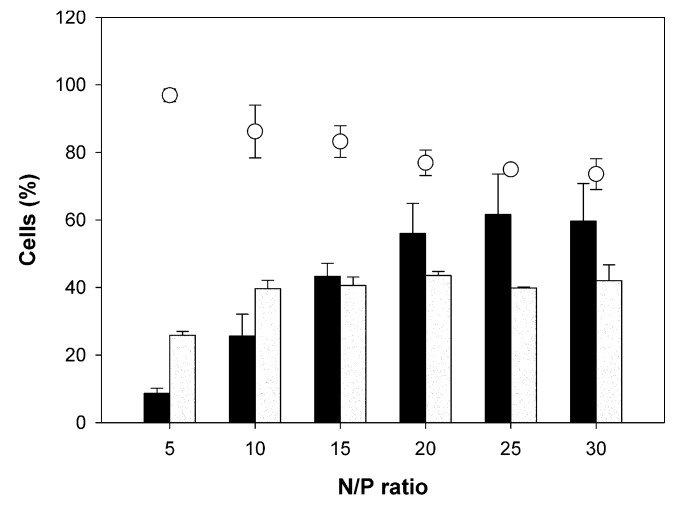
Transfection efficiencies (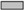), magnetic cell fractions (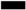), and viabilities (○) determined at 26 h post transfection. Data represent mean ± S.E.M., *n* ≥ 3.

Figure 9 compiles transfection efficiencies, viabilities and magnetic cell fractions obtained with DB. A transfection efficiency of 40% ± 3% was reached at N/P 10, together with an acceptable culture viability of 86% ± 7%.

**Figure 10:**
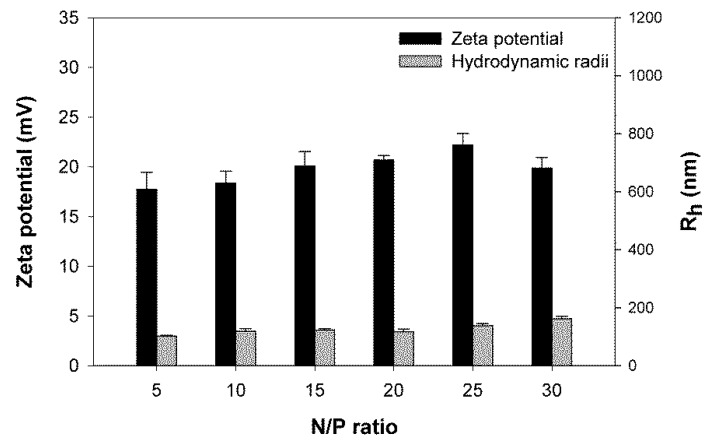
Average hydrodynamic radii (*R*_h_) and zeta potentials of polyplexes prepared with DB. Data represent mean ± S.E.M., *n* ≥ 3.

With a molecular weight (*M*_n_) of 975,000 kg/mol, the nano-star DB was by far the largest structure investigated. However, as shown in Figure 10, polyplexes formed by DB were not larger than those produced with some of the smaller nano-stars discussed above (see Figure 1 for comparison). We therefore excluded a simple size effect from being responsible for the slightly inferior performance of DB compared to P4/P5. Moreover, as Mendrek et al. showed, a high DEGMA content in the outer shell of PDMAEMA-based polymers is more toxic than a random distribution of DMAEMA and DEGMA units [30]. Preliminary testing with a second diblock nano-star with a similar arm number (91) and grafting density (0.035 arms/nm^2^), but significantly shorter PDMAEMA (540 instead of 1100) and PDEGMA (250 instead of 4350) blocks, showed improved transfection efficiency (66% ± 7%; N/P 20) and biocompatibility (94% ± 7%). At present, we presume that the low biocompatibility of DB is mainly due to the size of the PDEGMA block and that this also affects transfection efficiency.

**Figure 11:**
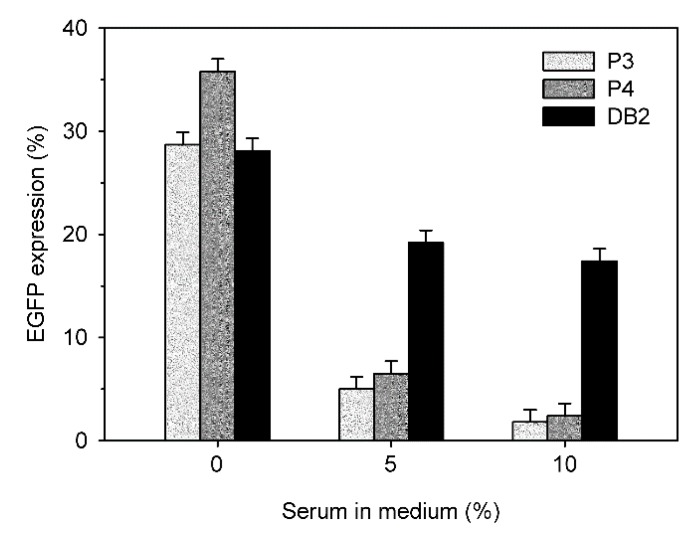
Effect of serum on transfection efficiencies of DB (N/P 20), P3 (N/P 20), and P4 (N/P 15). Data represent one experiment carried out in duplicate, with random experimental error shown.

A typical indication for the low blood compatibility of polycationic transfection agents is the loss of transfection efficiency in the presence of serum. Figure 11 compares the transfection efficiencies of P3, P4 and DB in the presence of 0%, 5%, and 10% fetal calf serum. The N/P ratios were individually chosen for each nano-star to assure comparable transfection rates and viabilities.

In all cases, a drop in transfection efficiency was observed with increasing serum concentration. However, this drop was much less pronounced for DB than for P3 and P4. In consequence, DB showed a four-fold higher transfection efficiency than P3 and P4 in the presence of 5% FCS, and a 6-fold higher transfection efficiency in the presence of 10% FCS.

**Figure 12:**
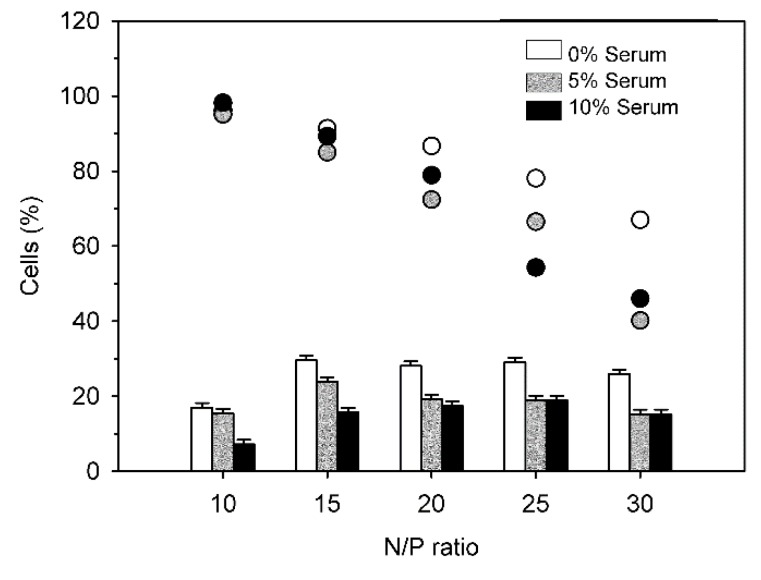
Transfection efficiency of DB in the presence of fetal calf serum as a function of the N/P ratio. Bars: percentage of EGFP-positive cells, circles: viabilities of cells. Data represent one experiment carried out in duplicate, with random experimental error shown.

Finally, Figure 12 shows transfection results with DB in presence of 0%, 5% and 10% serum as a function of the N/P-ratio.

Again, transfection efficiencies dropped somewhat in the presence of serum. This was observed for all investigated N/P-ratios. Interestingly, for N/P-ratios > 15, there was no statistically significant difference between transfection efficiencies in the presence of 5% and of 10% serum. However, increased serum levels exerted a negative effect on viabilities, especially at higher N/P ratios. No explanation can be given at present.

## 4. Conclusions

Transfection agents with magnetic properties enlarge the toolbox for studying non-viral gene delivery, since cellular magnetism is added as a new parameter. This allows, inter alia, a distinction between mere cellular interaction and actual uptake, which is otherwise difficult. Viability showed a much more pronounced dependency on the characteristics of the transfection agent/polyplex than the transfection efficiency itself, which should be taken into account during method optimization. End-capping the polycationic PDMAEMA arms with PDEGMA blocks improved the compatibility of the polycationic nano-stars with serum components. In consequence, such nano-stars may become good future candidates for in vivo applications. Preliminary data are available, showing that in order to maximize transfection efficiency and viability, a short PDEGMA block should be used.

## Figures and Tables

**Table 1:** Molecular characteristics of the nano-stars used in this study (sorted in ascending order of arm grafting density).

Designation	Arm number	Monomeric units		Core diameter (nm)	Grafting density (arms/nm²)	*M*_n_ (kg/mol)	PDI
		PDMAEMA block	PDEGMA block				
P1	5	300	-	16.5	0.006	235	1.2
P2	9	242	-	15.4	0.011	342	1.2
P3	20	528	-	15.4	0.024	1660	1.3
P4	46	1037	-	15.4	0.054	7498	1.6
P5	54	1470	-	15.4	0.064	12,470	1.4
P6	337	312	-	26.8	0.149	16,513	1.4
P7	411	477	-	26.8	0.182	30,825	1.5
P8	653	1240	-	26.8	0.289	127,335	1.5
P9	657	439	-	26.8	0.291	45,333	1.4
P10	679	1616	-	26.8	0.300	172,466	1.3
-	-	-	-	-	-	-	-
DB	98	1100	4350	28.8	0.038	975,000	1.6

PDMAEMA: poly(dimethylaminoethyl methacrylate). PDEGMA: poly(diethylene glycol) methyl ether methacrylate). PDI: polydispersity index (*M*_w_/*M*_n_).

**Table 2:** Amount of chemicals and reaction time required for synthesis of the nano-stars.

Designation	BIBSI (µL)	Particles (mg)	HMTETA (mg)	DMAEMA (g)	DEGMA (g)	Acetone (mL)	Reaction time(h)
P1	75	40	7.0	9.3	-	20	4
P2	75	50	8.3	18.7	-	20	2
P3	37.5	50	8.3	18.7	-	20	2
P4	37.5	50	8.3	18.7	-	20	4
P5	75	50	8.3	18.7	-	20	4
P6	36	40	7.0	23.3	-	25	4
P7	36	40	7.0	23.3	-	25	2
P8	75	40	7.0	23.3	-	25	4
P9	75	40	7.0	23.3	-	25	2
P10	75	40	7.0	23.3	-	25	8
-	-	-	-	-	-	-	-
DB	75	280 ^a^	27.0	-	117.3	100	48

^a^: γ-Fe_2_O_3_@Silica@PDMAEMA hybrid nanoparticles instead of γ-Fe_2_O_3_@Silica@BIBSI.

**Table 3:** Distribution of high, middle, and low producers within the EGFP-expressing cell fraction.

Fraction	Culture time post separation	Relative EGFP expression (%)		
		Low	Medium	High
control	0	16	47	37
non-magnetic	0	15	47	37
magnetic	0	25	44	32
control	25	15	28	57
non-magnetic	25	16	28	56
magnetic	25	14	26	60
control	49	14	37	49
non-magnetic	49	14	37	49
magnetic	49	12	33	55
